# Negative results: negative perceptions limit their potential for increasing reproducibility

**DOI:** 10.1186/s12952-015-0033-9

**Published:** 2015-07-07

**Authors:** Jaime A. Teixeira da Silva

**Affiliations:** P.O. Box 7, Miki-cho Post Office, Ikenobe 3011-2, Kagawa-ken, 761-0799 Japan

**Keywords:** COPE, Editorial bias, Negative results, STM publishers, Traditional peer review

## Abstract

Negative results are an important building block in the development of scientific thought, primarily because most likely the vast majority of data is negative, i.e., there is not a favorable outcome. Only very limited data is positive, and that is what tends to get published, albeit alongside a sub-set of negative results to emphasize the positive nature of the positive results. Yet, not all negative results get published. Part of the problem lies with a traditional mind-set and rigid publishing frame-work that tends to view negative results in a negative light, or that only tends to reward scientists primarily for presenting positive findings. This opinion piece indicates that in addition to a deficient mind-set, there are also severe limitations in the availability of publishing channels where negative results could get published.

There are, as I see it, two crises in science. The first relates to trust, in part, due to weaknesses and failures of the traditional peer review system [1]. The second, a crisis in reproducibility [2], is a knock-on effect of the first, partly because of lack of a widespread culture and acceptance of the need and importance of negative results.

Negative results are extremely important in science because they indicate what doesn’t work. Such valuable clues thus form the basis of new hypothesis testing and new experiments that could then allow a focus on a narrower set of variables, or options. The existence of negative results is an essential building block for science. Dr. Haiko Sprott defines a negative result as “a scientist is not able to show … a positive effect of the experiment”. [3]. Sandercock [4] provides a three-prong definition for negative results, including a third more somber, but valid, ethical perspective, within the context of studies involving human and animal subjects. These may be summarized as: 1) “truly inconclusive with ‘no evidence of effect’” (also referred to as neutral or uninformative results); 2) a study in which “any effect is too small to be worthwhile pursuing”; 3) “clear evidence of harm when benefit had been expected”.

Many, if not most, studies that show a set of data tend to present – most likely as an inherent human bias – the positive, successful results, in relation to negative results, either to show that the results are themselves positive, or to indicate that the negative ones were not successful. Thus, the intrinsic nature of many scientific papers already incorporates negative (or not so positive) results into its framework. Consequently, there are few outlets to publish purely negative results (Table 1) simply because: a) the majority of papers already cover a solid – but limited – selection of negative results, as explained above; b) mainstream science, technology and medicine (STM) publishers prefer to see a focus on the “positive” and not on the negative; c) scientists who would like to present only negative results might fear the equally negative perception by peers should they present only negative results. Related to c), the publication pressures that scientists face, and limits on time, cause them to set aside negative results in favor of positive ones in order to maximize their output, thus increasing scientific bias [5], and skepticism. This bias may lead science and scientists to know “more and more about less and less” [6]. Finally, excessive emphasis on the *P* value [7] inhibits authors from submitting results that are not significantly different while editors are skeptical about accepting results that either do not include statistical analyses, or that do not report significant differences. Adding to this complex background, McCormick [8] correctly points out one more limitation of the traditional peer reviewer pool: “the difficulty of finding reviewers canny enough to separate the null-result wheat from the ill-executed chaff”. Consequently, there might be a wealth of negative results with very positive messages and learning experiences that ought to be published to exploit novel avenues for new hypothesis testing.

**Table 1:** Journals that focus exclusively on negative results (listed alphabetically)

Journal name	Publisher	URL (start date^a^)
All Results Journals (Chem, Nano, Biol, Phys)*	SACSIS	http://arjournals.com/ (2010)
Journal of Articles in Support of the Null Hypothesis*	Reysen Group	http://www.jasnh.com/ (2002)
Journal of Errology	Unknown	http://www.bioflukes.com/ (unknown; discontinued; site down)
Journal of Interesting Negative Results	Unknown	http://jinr.site.uottawa.ca/ (2008; discontinued)
Journal of Negative Observations in Genetic Oncology	Unknown	http://www.path.jhu.edu/NOGO (1997; discontinued; site down)
Journal of Negative Results in BioMedicine*	BioMed Central (Springer Science + Business Medium)^a^	http://www.jnrbm.com/ (2002)
Journal of Negative Results – Ecology and Evolutionary Biology	JNR-EEB.org	http://jnr-eeb.org/index.php/jnr (2002; discontinued)
Journal of Pharmaceutical Negative Results*	Wolters Kluwer	http://www.pnrjournal.com/ (2010)
Nature Negative Results section*	Nature Publishing Group	http://www.nature.com/jcbfm/journal/v30/n7/full/jcbfm201051a.html (2010)^b^
The Journal of Spurious Correlations	International Sociology Association	http://www.jspurc.org/intro2.htm (2005; discontinued)
The International Journal of Negative & Null Results	International Journal Network	http://www.journalnetwork.org/journals/science-general/negative-null-results/negative-null-results.html (unknown; discontinued; site down)
The Null Journal	Unknown	http://null-journal.com/about.html (2009; discontinued)
University of Colorado Database of Negative Results	University of Colorado	https://sites.google.com/site/cujonr/ (2011; discontinued)

*Only these journals are still actively publishing (true up until June 29, 2015)

^a^In several cases, if the URL link is not working, or if details about the publisher or the archives could be found, or if no volumes had been published in at least one year, these journals were classified as “discontinued”.

^b^A special section of Journal of Cerebral Blood Flow & Metabolism, dedicated to negative results, was started in 2010. It is unclear if this section applies to other NPG journals.

So why then, apart from the negative psychological perception of negative results, are more negative results not published? There may also be two additional underlying factors, but ultimately these might be reflecting the negative aspects of positive psychology [9], which in terms of negative results in science, and their perception, are negatively viewed. As alluded to above, the first pertains to the limited selection of outlets (i.e., journals) in which such results could be published (Table 1). Even so, out of 13 journals that were initiated, only five remain active. Most mainstream STM publishers would most likely turn away a set of negative data results. This may also be associated with pride, as many/most STM journals wish only to showcase “the best” data sets, and, perhaps subconsciously, be actively downgrading the importance of negative data by not showcasing it. Other journals, especially those that continue to use a traditional print format, will prefer to accept positive results over negative results, i.e., an in-built editorial bias, associated with the psychology of the negative.

Consequently, the number of journals that can be found that deal exclusively with negative results is extremely limited (Table 1), most of which have been discontinued, and none of which carries an impact factor (IF). Unfortunately, the IF continues to serve – incorrectly – as a measure of quality [10], and many countries adopt a compensation-for-IF policy for their scientists, in which the latter are rewarded, sometimes monetarily, by publishing in IF journals. So, this vicious cycle of biased selection of IF journals, by scientists and their research institutes, automatically then tends to exclude the negative results. When there is no incentive by the “system” to embrace negative results, then these are also underplayed – if not totally ignored – by scientists themselves. The reality on the ground, i.e., the number of viable outlets for the publication of negative results (Table 1), is counter to the ethical basis that Sandercock [4] alludes to, namely that the results of human trials should be made publicly available, especially the negative results. His argument is that before any research project proposal is approved, it should reflect the entirety of the literature’s findings. Thus, if the scientific community has willfully ignored negative results, then not only does it represent a waste of tax-payer’s money, but also a valuable waste of resources (time, human effort, money, etc.) to discover what has already been discovered, but simply not reported, because there were insufficient, or inadequate, channels to demonstrate such negative data. Focusing on positive results by eliminating negative or unsuccessful options has particular relevance in R&D in the pharmaceutical industry, to reduce the waste of funds and to optimize resources [11]. Most likely as a result of these negative associations of not reporting negative results, reporting negative findings is now a requirement of the Consolidated Standards of Reporting Trials (CONSORT), specifically for clinical trials [12].

Curiously, the Committee on Publication Ethics (COPE) has one mandatory clause in its code of conduct for COPE member journal editors related to negative results that states: “14.3. Studies reporting negative results should not be excluded”. Thus, the importance of negative results is recognized, but they are simply not woven into the publishing psyche of scientists and editors or into the publishing fabric of most STM publishers. Thus, the mind set of editors, and their receptiveness towards negative results, needs an overhaul [13].

Most likely such a mental frame-work underlying the selection against negative in favor of positive ones will not only require a structural change, but also a mental or psychological one, including better training of editors to recognize the importance of negative results, and to distinguish negative results from bad science. Scientists also need to be taught to better appreciate the importance of their negative results, although this appreciation can only evolve when there are suitable and sufficient channels for them to express/publish their negative results. Providing additional and expansive ideas, through the public presentation of negative results, also aids in expanding the discussion, provides new vistas and perspectives, and assists those who wish to conduct similar experiments, with valuable experimental signs of what not to do, aptly summarized by Pfeffer and Olsen [14]: “it only takes one counter observation to falsify it”.

That said, the reader is duly warned that negative results should not be equated with bad science, lack of scientific rigor, or with poor experimental design. Negative results focus exclusively on those results that did not support a hypothesis, or prove a desired “positive” outcome, and should never be equated with, *senso lato*, bad science. The inflation of positive results, simply because there is a lack of negative ones, which have not been duly reported, may inflate the “positive” nature of some studies, and in some cases, these may turn out to be unreproducible. This was demonstrated in the Bayer HealthCare and Amgen cases, the latter not being able to replicate as much as 89 % of its published findings in prominent cancer journals, leading the National Institutes of Health (NIH) to contemplate the implementation of rules to validate positive results, and to counter the lack of incentives to publish negative results [15]. Thus, reporting negative results is one practical way to increase reproducibility. Journals that are luke-warm to the presentation of negative results could present a simple solution: the inclusion of a supplementary online file that summarizes the negative results. In fact, such a policy could or should be a standard practice. Such a solution would resolve the “gap” pointed out in a comment, made in [8], by Prof. Scott E. Kern, a Johns Hopkins pathologist: “If you sequence 13,000 genes, and only about 1,300 of them show mutations, then the other 11,700 sequences deposited are essentially null results”.

## Abbreviations

CONSORT: Consolidated Standards of Reporting Trials
COPE: Committee on Publication Ethics
IF: Impact factor
R&D: Research and development
STM: Science, technology and medicine
